# 

**Published:** 1899-12

**Authors:** 


### BOOKS RECEIVED.

Clinical Lectures on Neurasthenia. By Thomas D. Gavill, M. D., Physician to the West-End Hospital for Diseases of the Nervous System, London; Examiner for Clinical Medicine in the University of Glasgow; formerly Medical Superintendent of the Paddington Infirmary; Assistant Physician to the West London Hospital. Octavo, pp. 144. New York: William Wood & Co. 1899.

An Atlas of the Bacteria Pathogenic in Man. With descriptions of their Morphology and Modes of Microscopic Examination. By Samuel C. Shattuck, F. R. C. S., Joint Lecturer on Pathology and Bacteriology at St. Thomas's Medical School, London; Pathological Curator of the Museum of the Royal College of Surgeons, London. With an introductory chapter on bacteriology: its practical value to the general practitioner. By W. Wayne Babcock, M. D., Pathologist to the Kensington Hospital for Women; Clinical Pathologist to the Medico-Chirurgical Hospital, etc. Sixteen full-page colored plates. Price, $1.00. New York: E. B. Treat & Co. 1899.

Tenth Annual Report of the State Commision in Lunacy, October 1, 1897, to September 30, 1898. Transmitted to the legislature April 10, 1899. New York and Albany; Wynkoop-Hallenbeck-Crawford Co., State Printers. 1899. From Hon. Henry W. Hill, Member of Assembly, Buffalo.

Transactions of the American Surgical Association. Volume XVII. Edited by DeForest Willard, A. M., M. D., Ph. D., Recorder. Printed for the Association and for sale by William J. Dornan, Philadelphia. 1899.

Collins and Rockwell's Physiology. Just ready. A Pocket Text-Book of Physiology. By H. D. Collins, M. D., Assistant Demonstrator of Anatomy, and W. H. Rockwell, Jr., A. B, M. D., Assistant Demonstrator of Anatomy, College of Physicians and Surgeons, New York. In one handsome 12mo volume of 316 pages with 153 illustrations. Cloth, $1.50 net.

The Urine and the Clinical Chemistry of the Gastric Contents, the Common Poisons and Milk. By J. W. Holland, M. D., Professor of Medical Chemistry and Toxicology, Jefferson Medical College of Philadelphia. Sixth edition, revised and enlarged. Duodecimo, forty-one illustrations. Cloth, price, $1.00 net. Philadelphia: P. Blakiston's Son & Co., 1012 Walnut Street.

Twentieth Century Practice. An International Encyclopedia of Modern Medical Science. By leading authorities of Europe and America. Edited by Thomas L. Stedman, M. D., New York City. In twenty volumes. Volume XVIII. Syphilis and Leprosy. New York: William Wood & Co. 1899.

A Practical Treatise on Materia Medica and Therapeutics. By Roberts Bartholow, M. A., M. D., LL. D., Professor Emeritus of Materia Medica, General Therapeutics and Hygiene in the Jefferson Medical College of Philadelphia; formerly Professor of Materia Medica and Therapeutics and of the Practice of Medicine in the Medical College of Ohio, etc., etc. Tenth edition, revised and enlarged. Octavo, pp. xxii.—866. New York: D. Appleton & Co. 1899.

Essentials of Diseases of the Skin Including the Syphilodermata. Arranged in the form of questions and answers, for students of medicine. By Henry W. Stelwagon, M. D., Ph. D., Clinical Professor of Dermatology in the Jefferson Medical College; Physician to the Department of Skin Diseases in Howard Hospital, etc. Fourth edition, revised and illustrated. Saunders's Question Compends No. 11. Price, $1.00. Philadelphia: W. B. Saunders. 1899.

Essentials of Medical Chemistry, Organic and Inorganic. Containing questions of Medical Physics, Chemical Philosophy, Analytical Processes, Toxicology, etc. Arranged for students of Medicine. By Lawrence Wolff, M. D., Demonstrator of Chemistry, Jefferson Medical College; Physician to the German Hospital, etc., etc. Fifth edition, revised by Smith Ely Jelliffe, M. D., Ph. D., Professor of Pharmacognosy, College of Pharmacy, New York, etc. Saunders's Question Compends No. 4. Price, $1.00. Philadelphia: W. B. Saunders. 1899.

Essentials of Anatomy Including the Viscera. Arranged in the form of questions and answers, for students of medicine. By Charles B. Nancrede. M. D., Professor of Surgery and Clinical Surgery in the University of Michigan; Emeritus Professor of General and Orthopedic Surgery, Philadelphia Polyclinic, etc. Sixth edition, revised by Fred J. Brockway, M. D., Assistant Demonstrator of Anatomy, Columbia University, New York. Saunders's Question Compends No. 3. Price, $1.00. Philadelphia: W. B. Saunders. 1899.

Essentials of Physical Diagnosis of the Thorax. By Arthur M. Corwin, A. M., M. D., Instructor of Physical Diagnosis at Rush Medical College; Attending Physician to the Central Free Dispensary, etc. Third edition, revised and enlarged. Price, 1.00. Philadelphia: W. B. Saunders. 1899.

Warner's Pocket Medical Dictionary of Today. Comprising pronunciation and definition of 10,000 essential words and terms used in medicine and associated sciences, and tables of arteries, nerves, muscles, etc. Arranged for convenient reference. By William R. Warner. Copyright. Price, 75 cents. Philadelphia: William R. Warner & Co. 1899.

Lectures upon the Principles of Surgery. Delivered at the University of Michigan. By Charles B. Nancrede, A. M., M. D., LL. D., Professor of Surgery and Clinical Surgery; Emeritus Professor of General and Orthopedic Surgery, Philadelphia Polyclinic, etc., etc. With an appendix containing a résumé of the principal views held concerning inflammation. By William A. Spitzley, A. B., M. D., Senior Assistant in Surgery, University of Michigan. Illustrated. Price, $2.50. Philadelphia: W. B. Saunders. 1899.

A Text-Book of Embryology for Students of Medicine. By John Clement Heisler, M. D., Professor of Anatomy in the Medico-Chirurgical College, Philadelphia. With 190 illustrations, of which twenty-six are in colors. Price, $2.50. Philadelphia: W. B. Saunders. 1899.

Operative Surgery. By Joseph D. Bryant, M. D., Professor of the Principles and Practice of Surgery, Operative and Clinical Surgery, University and Bellevue Hospital Medical College; Visiting Surgeon to Bellevue and St. Vincent's Hospitals; Consulting Surgeon to the Hospital for Ruptured and Crippled, Woman's Hospital and Manhattan State Hospital, etc. Volume I. General Principles, Anesthetics, Antiseptics, Control of Hemorrhage, Treatment of Operation-Wounds, Ligature of Arteries, Operations on Veins, Capillaries, Nervous System, Tendons, Ligaments, Fasciæ, Muscles, Bursæ and Bones, Amputations, Deformities, Plastic Surgery. This volume contains 749 illustrations, forty-nine of which are colored. New York: William Wood & Co. 1899.

The Modern Treatment of Wounds. By John E. Summers, Jr., M. D., Surgeon-in-Chief to the Clarkson Memorial Hospital; Attending Surgeon Douglas County Hospital; formerly Professor of the Principles and Practice of Surgery and Clinical Surgery, Omaha Medical College; Ex-President of the Western Surgical and Gynecological Association, the Nebraska State Medical Society, and the Omaha Medical Society. Omaha: Medical Publishing Co., Publishers. 1899. Price $1.50.

The Surgical Diseases of the Genito-urinary Tract, Venereal and Sexual Diseases. A text-book for students and practitioners. By G. Frank Lydston, M. D., Professor of the Surgical Diseases of the Genito-Urinary Organs and Syphilology in the Medical Department of the State University of Illinois; Professor of Criminal Anthropology in the Kent College of Law; Surgeon-in-Chief of the Genito-Urinary Department of the West-Side Dispensary. Fellow of the Chicago Academy of Medicine; Fellow of the American Academy of Political and Social Science; Delegate from the United States to the International Congress for the Prevention of Syphilis and the Venereal Diseases, held at Brussels, Belgium, September 5, 1899, etc. Illustrated with 233 engravings. 6½ x 9¾ inches. Pages xvi–1024. Extra Cloth, $5.00, net; sheep or half-Russia, $5.75 net. Philadelphia. The F. A. Davis Co., Publishers, 1914-16 Cherry St.

Bacteriology in Medicine and Surgery. A practical manual for physicians, health officers and students. By William H. Park, M. D., Associate Professor of Bacteriology and Hygiene in the University and Bellevue Hospital Medical College, New York In one 12mo volume, 688 pages, with 87 illustrations in black and colors, and two full-page colored plates. Just ready. Cloth, $3.00 net.

Annual and Analytical Cyclopedia of Practical Medicine. By Charles E. de M. Sajous, M. D., and 100 associate editors. Assisted by corresponding editors, collaborators and correspondents. Illustrated with chromo-lithographs, engravings and maps. Volume IV. Infants, diarrheal diseases of, to mercury. Philadelphia, New York and Chicago: F. A. Davis Company. 1899.

A Text-Book of Materia Medica, Therapeutics and Pharmacology. By George Frank Butler, Ph. G., M. D., Professor of Materia Medica and of Clinical Medicine in the College of Physicians and Surgeons, Medical Department, University of Illinois, Chicago; Professor of General Medicine and Diseases of the Digestive System, Chicago Clinical School; Attending Physician to Cook County Hospital, etc., etc. Third edition, thoroughly revised. Handsome 8vo volume of 874 pages. Illustrated. Philadelphia: W. B. Saunders. 1899.

Treatise on Orthopedic Surgery. By Edward H. Bradford, M. D., Surgeon to the Children's Hospital and the Samaritan Hospital; Assistant professor of Orthopedic Surgery in Harvard Medical School; and Robert W. Lovett, M. D., Assistant Surgeon to the Children's Hospital; Surgeon to the Infants' Hospital. Illustrated with 621 engravings. Second revised edition. New York: William Wood & Company. 1899.

A Text-Book of the Practice of Medicine. By James M. Anders, M. D., Ph. D., LL. D., Professor of the Practice of Medicine and of Clinical Medicine in the Medico-Chirurgical College, Philadelphia; Attending Physician to the Medico-Chirurgical and Samaritan Hospitals, Philadelphia, etc. Octavo, pp. 1292. Illustrated. Third edition, revised. Price, cloth, $5.50; sheep, $6.50. Philadelphia: W. B. Saunders, 925 Walnut Street. 1899.

An American Text-Book of Surgery. For practitioners and students. By Phineas S. Conner, M. D., Frederic S. Dennis, M. D., William W. Keen, M. D., Charles B. Nancrede, M. D., Roswell Park, M. D., Lewis S. Pilcher, M. D., Nicholas Senn, M. D., Francis J. Shepherd, M. D., Lewis A. Stimson, M. D., J. Collins Warren, M. D., and J. William White, M. D. Edited by William W. Keen, M. D., L.L. D., and J. William White, M. D., Ph. D. Third Edition Thoroughly revised. Imperial 8vo pp. xvi.—1228. Price, $7.00 cloth; $8.00 sheep, and $9.00 half-Russia. Philadelphia: W. B. Saunders, 925 Walnut Street. 1899.

---

RECENT mail advices from Manila received at the War Department show that General Otis has established a medico-legal department in Manila, in charge of two Filipino physicians, José R. Hidalgo and Gregorio Singlan. An emergency ward and dissecting-room has also been established for post mortem examinations. The department is to be subject to the orders of the Supreme Court and the tribunals of justice in the city of Manila.

